# Alcohol, Tuberculosis, and the Public-House

**Published:** 1902-09-20

**Authors:** 


### ALCOHOL, TUBERCULOSIS, AND THE PUBLIC-HOUSE.

The remarks made at the Tuberculosis Congress last year by Professor Brouardel in regard to the large part played by alcoholism in the production of tuberculous disease drew attention very strongly to an aspect of the question which many had overlooked, and at the same time, no doubt, led some people to draw inferences as to the virulent action of alcohol as a predisponent to tuberculosis which went a good deal beyond what could be supported by evidence. The gross fact of the association remained, and the Professor's remarks served well to emphasise the view which had been long taken by many observers that, so far as statistics were available, one of the most potent influences in predisposing to phthisis was drink. Still, though it was well known that alcoholism produced catarrh and hæmorrhage, and might be regarded as one of the many causes by which those injuries of the lung are produced which render these organs liable to the deposit of tubercle, it was by no means clear that the frequency or extent of this form of injury was such as to account for the very common association of the two conditions; and the thanks of all interested in the matter are due to Dr. Niven, of Manchester, for the suggestion, and for the trouble he has taken to support the suggestion, that it is from the place where the drink is taken, namely, the public-house, with its dirty, dusty floor on to which spitting takes place all day long, that the infection of tuberculosis is derived. He points out that in many forms of industry associated with an excessive phthisis mortality, industries in which this association might be presumed to arise from the heat, the dust, or the evil fumes concerned, the habit of spitting prevails, and that this may not improbably be the influence which leads to the association of these industries with tuberculosis. And so with the public-house. It is here that the well-paid and well-lodged artisan comes in intimate contact, so far as regards breathing the same dust-laden air, with the poor, ill-fed, ill-lodged, and often tuberculous labourer. It is here that they all spit promiscuously upon a floor which is but seldom thoroughly cleaned, and the dust from which is kept in a constant state of agitation, and it is here that while imbibing the alcohol (which may in a certain degree injure the lungs just as it may injure them when taken under better conditions) he at the same time takes in his daily dose of dust infected by the expectoration of a promiscuous crowd always recruited more or less from the lodging-house class of tuberculous labourers. Both the customers and the servants of the public-house suffer unduly from tuberculosis, partly, no doubt, because of their exposure to alcohol, but far more because the public-house floor is constantly spat upon, and the public-house dust is as constantly tuberculous. It is an interesting deduction. It would appear that in early life females suffer most from tuberculosis, probably from their being affected in a larger degree than males by tuberculous home influences; but in later years some other influence comes into play which makes the males suffer more than females, and although that influence may be alcohol, it is perhaps still more the public-house, the dirty tubercle-infected place in which the alcohol is consumed.

